# The efficacy and safety of beinaglutide alone or in combination with insulin glargine in Chinese patients with type 2 diabetes mellitus who are inadequately controlled with oral antihyperglycemic therapy: A multicenter, open‐label, randomized trial

**DOI:** 10.1111/1753-0407.13483

**Published:** 2023-10-20

**Authors:** Xiangyang Liu, Wenjuan Yang, Jianrong Liu, Xinxi Huang, Yujie Fang, Jie Ming, Jingbo Lai, Jianfang Fu, Qiuhe Ji, Li Wang

**Affiliations:** ^1^ Department of Endocrinology, Xijing Hospital Air Force Medical University Xi'an China; ^2^ Department of Endocrinology Shaanxi Aerospace Hospital Xi'an China; ^3^ Department of Endocrinology Xi'an Chang An Hospital Xi'an China

**Keywords:** beinaglutide, fasting C‐peptide, insulin glargine (IGlar)

## Abstract

**Background:**

To compare glycemic control in Chinese patients with type 2 diabetes mellitus (T2DM) whose blood glucose levels were inadequately controlled with oral antidiabetic drugs after beinaglutide alone or combined with insulin glargine (IGlar).

**Methods:**

In this 16‐week multicenter, randomized clinical trial, 68 participants randomly received beinaglutide or IGlar for 8 weeks, then the two drugs in combination for 8 weeks. The primary outcomes were the proportion of individuals achieving their glycemic target and the change in glucose variability as measured with a continuous glucose monitoring system from baseline to 8 and 16 weeks.

**Results:**

Both the beinaglutide and IGlar groups showed increased proportions achieving their glycemic target at 8 weeks, and the combination augmented the proportion reaching the glycated hemoglobin target from 25.42% at 8 weeks to 40.68% at 16 weeks. The beinaglutide group showed a significant reduction in body weight, body mass index, waist circumference, and systolic blood pressure. Beinaglutide elevated high‐density lipoprotein cholesterol by 0.08 mmol/L (95% confidence interval [CI], 0.00–0.16), and diminished low‐density lipoprotein cholesterol by 0.21 mmol/L (95% CI, 0.05–0.48), whereas IGlar showed no effect. Though IGlar was more efficient in lowering fasting plasma glucose than beinaglutide at comparable efficacies (to −1.57 mmol/L [95% CI, −2.60 to −0.54]), this difference was abolished in patients whose fasting C‐peptide was ≥0.9 ng/mL.

**Conclusion:**

Beinaglutide exhibited a favorable hypoglycemic effect on patients with T2DM, and in combination with IGlar, glucose level was further decreased. Low fasting C‐peptide in patients may reduce the glycemic response to beinaglutide therapy. We recommend that C‐peptide levels be evaluated when using or switching to the novel glucagon‐like peptide‐1 receptor agonists beinaglutide.

**Trial Registration:**

ClinicalTrials.gov: NCT03829891.

## Graphical abstract

**Highlights**

- This was the first‐ever multicenter, open‐label, randomized trial to compare the hypoglycemic efficacy and safety of beinaglutide versus insulin glargine (IGlar) or beinaglutide combined with IGlar.
- Beinaglutide combined with IGlar attenuated fasting plasma glucose further and improved the proportion of patients with type 2 diabetes mellitus (T2DM) reaching their glycemic target relative to beinaglutide alone.
- Beinaglutide reduced systolic blood pressure and improved lipid parameters in patients with T2DM.
- The hypoglycemic outcome with beinaglutide was related to the patients' baseline fasting C‐peptide.

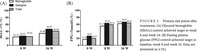

## 1 INTRODUCTION

Type 2 diabetes mellitus (T2DM) is a complex metabolic disorder, and because of its progressive nature, most patients with T2DM eventually require combined drug treatment that can be in the form of antihyperglycemic agents administered as either oral or injectable therapies. The choice of combination drug therapy is based on the clinical characteristics of patients and their preferences, other comorbidities, and specific adverse drug effects as well as safety, tolerability, and cost.^1^

In general, when oral antidiabetic drug regimens cannot lower glycated hemoglobin (HbA1c) to the targeted value, adding basal insulin (BI) is a widely adopted combination therapy.^2^, ^3^ BI is considered the most convenient and effective initial insulin regimen for patients with poor glycemic control. Approximately one third of Chinese patients with diabetes use insulin for antihyperglycemic treatment, of whom 26.7% use BI alone and 13.4% use BI combined with prandial insulin.^4^, ^5^ As a long‐acting insulin analog, insulin glargine (IGlar) is characterized by a longer duration of action and with a less‐pronounced peak, effectively reducing HbA1c and fasting plasma glucose (FPG); the risk of hypoglycemia, especially nocturnal hypoglycemia, is significantly lower than with neutral protamine Hagedorn insulin.^6^, ^7^ The regimen is administered once per day to allow ease of use and to comply with the needs of those patients who require assistance with insulin injection; IGlar is the most commonly used BI.^8^

Glucagon‐like peptide‐1 receptor agonists (GLP‐1 RAs) can effectively lower blood glucose in a glucose‐dependent manner, without an increased risk of hypoglycemia.^9^, ^10^ These drugs have also been shown to reduce body weight and reduce the risk of atherosclerotic cardiovascular disease (ASCVD).^11^, ^12^, ^13^ Many guidelines recommend GLP‐1 RAs as the preferred drug for patients with ASCVD or for indications of high ASCVD risk, chronic kidney disease or heart failure independent of HbA1c or metformin use.^1^, ^14^, ^15^

A narrative review of head‐to‐head comparisons showed that for glucose control, short‐acting GLP‐1RAs exerted a greater impact on postprandial plasma glucose (PPG) levels compared with continuous‐acting GLP‐1RAs, but for FPG and HbA1c, continuous‐acting treatments demonstrated a greater effect.^16^ There are relatively few studies on the clinical efficacy and safety of beinaglutide, a short‐acting GLP‐1RA. It is a fully humanized GLP‐1 RA (recombinant human glucagon peptide‐1 [7–36], with an amino acid sequence of His‐Ala‐Gly‐Thr‐Phe‐Thr‐Ser‐Asp‐Val‐Ser‐Ser‐Tyr‐Leu‐Glu‐Gly‐Gln‐Ala‐Ala‐Lys‐Glu‐Phe‐IIe‐Ala‐Trp‐Leu‐Val‐Lys‐Gly‐Arg. The molecular formula of beinaglutide is C149H225N39O46, and it has a molecular weight of 3298.7 kDa. Laboratory studies show that beinaglutide inhibits high fat diet‐induced obesity by targeting the composition of major lipid classes and the expression of genes in lipid metabolism of adipose tissues in mice.^17^

With a half‐life of 11 min, beinaglutide is currently administered as a subcutaneous injection three times per day, 5 min before meals, In a clinical study, investigators showed that beinaglutide effectively lowered HbA1c, FPG, and PPG.^18^ Compared with sulfonylureas, beinaglutide provided similar reductions in HbA1c but lowered the incidence of hypoglycemia.^19^ It also manifested a favorable effect on weight loss.^20^ The use of beinaglutide in obese nondiabetic patients showed that its use was beneficial for weight loss, particularly pertaining to visceral fat.^20^, ^21^ Beinaglutide is also able to improve vascular endothelial function.^22^

In a meta‐analysis, the once‐weekly GLP‐1 RAs, exenatide long‐acting release and dulaglutide, led to greater HbA1c reductions versus basal insulins, whereas once‐daily liraglutide and twice‐daily exenatide did not.^23^ However, BI increased hypoglycemic risk compared with GLP‐1RAs,^24^ and compared with IGlar, treatment with GLP‐1 RAs resulted in greater weight loss but increased adverse gastrointestinal events.^25^, ^26^ As there are currently few head‐to‐head studies to evaluate the comparative hypoglycemic effect between beinaglutide and BI, we herein conducted a study to observe the efficacy and safety of beinaglutide versus IGlar with respect to glycemic regulation in inadequately controlled T2DM patients. We also analyzed the efficacy of the combination of beinaglutide and glargine to explore the advantages of their combined use.

## 2 METHODS

### 2.1 Study design and ethical compliance

This was an investigator‐initiated, multicenter trial that consisted of a screening period; 0–8 week randomized, open‐label treatment period with beinaglutide or IGlar; and a 9–16 week treatment period that encompassed treatment for those patients who reached their targets or combination therapy with beinaglutide and IGlar. Three study sites participated in the trial. This study was designed and conducted according to the Good Clinical Practice guidelines and was approved by the independent ethics committee or institutional review board at each center before the study began. This study is registered with ClinicalTrials.gov under accession number NCT03829891.

### 2.2 Study participants

Participants were aged between 18 and 70 years at screening, with confirmed T2DM ≥6 months prior to the study. Study candidates consisted of men and nonpregnant women using a medically approved birth‐control method who were treated with a stable hypoglycemic drug (alone or in combination therapy, excluding glinides, dipeptidyl‐peptidase‐VI inhibitors, insulin, and GLP‐1 agonists) for ≥1 month, with HbA1c ≥7.5% and ≤11.0% at screening or within 2 weeks before screening and with a body mass index (BMI) of 21–35 kg/m^2^.

Exclusion criteria included those with a clinically significant medical condition that precluded safe participation in the study; women with childbearing potential who did not use an eligible contraceptive throughout the study or were breastfeeding; women undergoing treatment with systemic corticosteroids for ≥7 days; those with fasting triglycerides >4.5 mmol/L; those with a history of allergy (such as systemic anaphylaxis, angioneuroedema, or epidermal exfoliation), a history of organ transplantation or AIDS; a history of medullary thyroid carcinoma, or a history of alcohol or drug abuse and individuals at potential risk of noncompliance with our protocol, as well as individuals judged by the investigator to be unsuitable for this study.

### 2.3 Intervention

**FIGURE 1:**
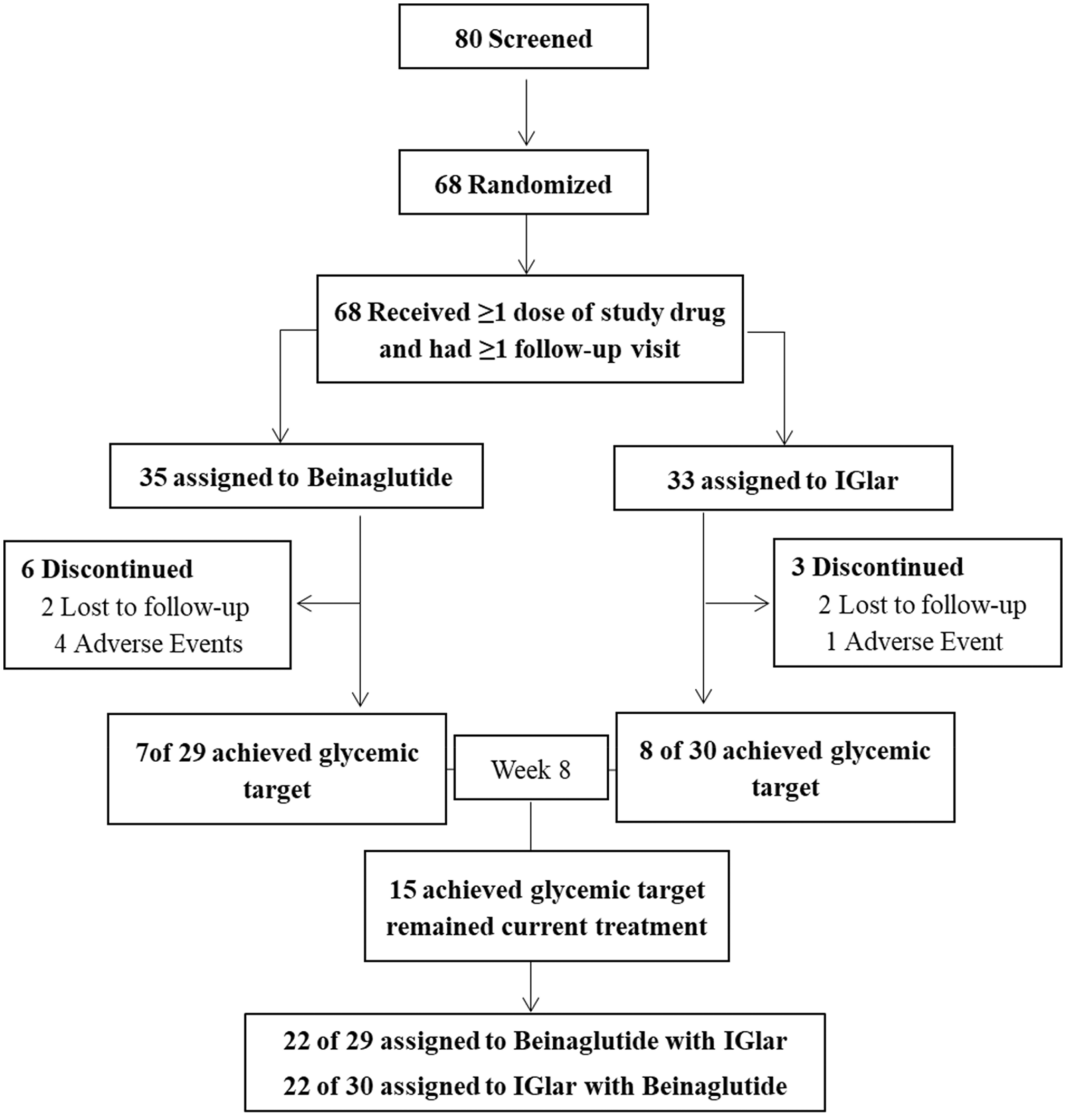
Flow of participants through the study. Full analysis set (FAS), including all randomized subjects receiving at least one dose of any of the trial products. Per‐protocol set (PPS), which was the secondary population for efficacy evaluation in this study. IGlar, insulin glargine.

All eligible participants were randomized at a ratio of 1:1 to one of the two treatments (beinaglutide or IGlar) through a central randomization system using an interactive voice/web response system, and this study included a 1‐week screening period during which participants received their previous treatment. Patients in the beinaglutide group received 0.1 mg of beinaglutide (Shanghai Renhui Biopharmaceutical Co., Ltd., China) via subcutaneous injection three times per day, 5 min before breakfast, lunch, and dinner during the first weeks of the treatment and then received 0.2 mg of beinaglutide three times per day during the next weeks. Participants in the IGlar group received IGlar (Lantus U100, Sanofi; Gentilly, France) via subcutaneous injection starting at a dose of 0.2 IU/kg or 10–12 IU/day before bed. Doses of IGlar were titrated to a target prebreakfast fasting glucose of <7.0 mmol/L based on self‐measured blood glucose values 3 days prior to the visit. We conducted insulin‐dose adjustments on an individual basis to achieve optimal glycemic control (target fasting plasma glucose [FPG] <7 mmol/L) and to minimize the risk of hypoglycemia as dictated by clinical practice. At the end of 8 weeks, enrollees who reached optimal glucose control (FPG <7.0 mmol/L and HbA1c <7%) remained on their current treatment, and the other participants who could not meet their glycemic target were administered a combination of beinaglutide and IGlar (Figure 1).

### 2.4 Outcomes

The primary efficacy end points were the proportion of individuals achieving their glycemic target (ie, FPG <7 mmol/L and HbA1c <7%), and the changes in glucose variability as measured with a continuous glucose monitoring system at week 8 or 16 from baseline, including mean glycemic (MG), mean amplitude of glycemic excursions (MAGE), large amplitude of glycemic excursions (LAGE), SD of blood glucose (SDBG), and mean of daily differences (MODD). The secondary efficacy end points were the changes from baseline to 8 and 16 weeks of treatment in (1) FPG and 2‐h PPG after a standard meal; (2) blood pressure; (3) lipid metabolism, including total cholesterol, low‐density lipoprotein cholesterol (LDL‐C), high‐density lipoprotein cholesterol (HDL‐C), and triglycerides (TG); (4) body weight and BMI; (5) inflammatory factors, including 8‐iso‐prostaglandin F_2α_ (8‐iso‐PGF2α), monocyte chemotactic protein‐1 (MCP‐1), and high sensitive C‐reactive protein (Hs‐CRP), and (6) urinary albumin.

To evaluate the safety and tolerability of the two drugs, adverse events (AEs), including serious adverse events (SAEs) and reported hypoglycemia events (patient‐reported symptoms of hypoglycemia that were promptly resolved after food intake or intravenous glucose); identified hypoglycemia (a blood glucose level ≤3.9 mmol/L regardless of whether there were symptoms of hypoglycemia); vital signs and clinical biochemical and hematological parameters; and electrocardiogram data were recorded using standard methods.

### 2.5 Statistical analyzes

Sixty‐eight participants were each randomized at a ratio of 1:1 to one of the two treatment groups for this trial. This sample size was determined using estimates generated by NCSS PASS 11 (NCSS LLC, Kaysville, UT, USA) software. To compare the hypoglycemic effects of beinaglutide and IGlar, we calculated the sample size based on our clinic's data and previous studies.^27^ We determined that a sample size of 29 patients per group was necessary, for a two‐sided 0.05 significance level and a power of 80%. With a dropout rate of 15%, a total of 68 patients were recruited. The final sample size was determined to be 35 for the beinaglutide group and 33 for the IGlar group. Only those participants who received the assigned treatment continuously throughout the entire process were included in our analysis. The per‐protocol set (PPS) comprised all randomized participants who completed the study with no major protocol violations that affected study outcomes, and the PPS was then used for supportive analyses. Demographic characteristics and safety analyses were conducted on all randomized participants who received ≥1 dose of study drug and who possessed baseline safety data and safety data for at least one follow‐up visit.

We adopted SPSS 19.0 software (SPSS Inc., Chicago, IL, USA) to conduct all statistical analyses. Quantitative indices are presented as mean ± SD, or median and interquartile; qualitative indices are recorded using a frequency distribution table or percentage. Two‐sided tests were used in all cases, and *p* < .05 was considered to be statistically significant. Fisher's exact‐probability test was applied to compare the attrition rates between the groups. We executed the chi‐square test or Fisher exact probability test (if the frequency was less than 5) to evaluate the differences between categorical variables before and after treatment within groups; the paired sample *t* test or Wilcoxon signed‐rank test was used for continuous variables. To analyze the changes among groups, analysis of covariance was used with baseline values as covariates and treatment site as the fixed factor and presented as least‐square (LS) means with a corresponding Dunnett 95% confidence interval (95% CI), and the Dunnett LS mean differences (95% CI) test was implemented to assess differences between the groups. The least‐square mean and 95% CI of the difference before and after treatment in each group were calculated, respectively, and we judged whether there was a significant change after treatment according to whether the 95% CI included 0. Incidence rates of adverse events for the two groups were compared with Fisher's exact test. Statistical significance was accepted at *p* < .05.

## 3 RESULTS

### 3.1 Participant demographics and baseline characteristics

Of the 80 participants screened, 68 were randomized to receive beinaglutide (the beinaglutide group) or IGlar (the IGlar group). Six participants in the beinaglutide group and three in the IGlar group withdrew from the study. Fifty‐nine (86.76%) participants ultimately completed the study. The full analysis set included 68 participants (35 and 33 in the beinaglutide and the IGlar groups, respectively), and the PPS included 59 participants (29 and 30 in the beinaglutide and IGlar groups, respectively) (Figure 1).

Men comprised 72% of the participants. Mean weight was 69.73 kg, BMI was 24.71 kg/m^2^, mean waist circumference was 89.93 cm, and mean HbA1c was 9.18% in the beinaglutide group. In the IGlar group, mean weight was 72.36 kg, BMI was 24.87, mean waist circumference was 93.58 cm, and mean HbA1c was 9.24%. There were no significant differences in demographics or baseline characteristics between the two groups (Table 1).

**TABLE 1:** Patient demographics and baseline characteristics (full analysis set).

Variable	Beinaglutide (*N* = 35)	IGlar (*N* = 33)	*p* value
Male, *n* (%)	23 (65.7)	26 (78.8)	.285
Age, years	52.6 ± 11.55	53.3 ± 8.13	.755
Weight, kg	69.73 ± 14.45	72.36 ± 10.57	.179
Height, cm	167.33 ± 8.11	170.06 ± 7.61	.157
BMI, kg/m^2^	24.71 ± 3.44	24.87 ± 2.48	.354
Waist circumference, cm	89.93 ± 9.87	93.58 ± 6.82	.190
Pulse, beats/min	77.4 ± 7.42	78.1 ± 7.19	.130
SBP, mm Hg	121.1 ± 13.61	121.3 ± 12.47	.627
DBP, mm Hg	75.7 ± 7.64	75.5 ± 7.95	.370
HbA1c, %	9.18 ± 1.06	9.24 ± 0.94	.288
FPG, mmol/L	8.88 ± 2.21	8.78 ± 2.74	.775
Two hour PPG, mmol/L	16.17 ± 3.20	16.24 ± 3.78	.633
Fasting serum insulin, μIU/mL	7.21 ± 3.91	6.86 ± 5.22	.623
Fasting serum C‐peptides, ng/mL	1.52 ± 0.73	1.47 ± 0.65	.639
Glucagon, pmol/L	5.69 ± 5.12	6.39 ± 5.48	.715
TC, mmol/L	4.50 ± 0.94	4.57 ± 1.14	.205
HDL‐C, mmol/L	1.18 ± 0.26	1.22 ± 0.24	.217
LDL‐C, mmol/L	2.82 ± 0.83	2.80 ± 0.91	.552
TG, mmol/L	1.87 ± 0.92	2.00 ± 1.04	.847
Uric acid, μmol/L	299.34 ± 77.42	292.98 ± 78.02	.789
8‐Iso‐PGF2α, ng/mL	208.34 (136.27–405.27)	253.79 (165.09–389.11)	.660
MCP‐1, ng/mL	192.61 (146.51–246.06)	193.61 (157.24–229.11)	.873
CRP, mg/dL	829.38 (240.71–2219.39)	674.43 (410.16–1286.64)	.782
Urinary albumin, mg/L	18.1 (9.81–32.85)	10.8 (7.1–50.3)	.506
MG, mmol/L	10.34 ± 1.70	10.36 ± 2.47	.198
MAGE, mmol/L	4.71 ± 1.46	4.28 ± 1.51	.722
LAGE, mmol/L	7.54 ± 1.94	7.54 ± 1.94	.586
MODD, mmol/L	2.61 ± 0.64	2.61 ± 0.64	.996
SDBG, mmol/L	2.57 ± 1.91	2.57 ± 1.91	.457

*Note*: Data are presented as *n* (%), mean (SD), or median (range interquartile) in patients who received ≥1 dose of study drug and had at least one follow‐up visit. *p* value: comparison between the groups.

Abbreviations: 8‐Iso‐PGF2α, 8‐iso‐prostaglandin F2α; two‐hour PPG, 2 h postprandial plasma glucose; BMI, body mass index; CRP, C‐reactive protein; DBP, diastolic blood pressure; FPG, fasting plasma glucose; HbA1c, glycated hemoglobin; HDL‐C, high‐density lipoprotein cholesterol; IGlar, insulin glargine; LAGE, the largest amplitude of glycemic excursions; LDL‐C, low‐density lipoprotein cholesterol; MAGE, mean amplitude of glycemic excursions; MCP‐1, monocyte chemoattractant protein‐1; MG, mean glycemic; MODD, mean of daily difference; SBP, systolic blood pressure; SDBG, SD of blood glucose; TC, total cholesterol; TG, triglycerides.

### 3.2 Proportion of patients achieving their glycemic targets in HbA1c and FPG

**FIGURE 2:**
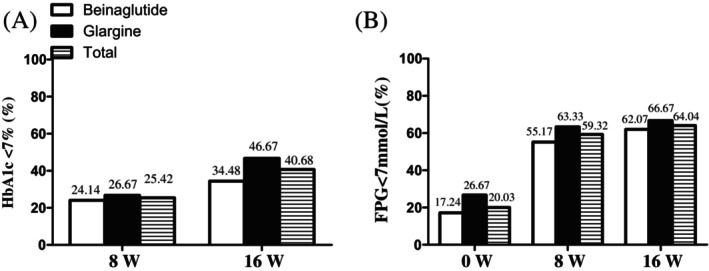
Primary end points after treatments. (A) Glycated hemoglobin (HbA1c) control achieved target at week 8 and week 16. (B) Fasting plasma glucose (FPG) control achieved target at baseline, week 8 and week 16. Data are presented as *n* (%).

The proportion of individuals with an HbA1c <7% was 24.14% with beinaglutide versus 26.67% with IGlar, with no statistical differences between the two groups (*p* = 0.753) at week 8. From week 8 to week 16, patients taking beinaglutide combined with IGlar who did not reach their glucose target and those with an HbA1c of <7% comprised 34.48% of the beinaglutide group and 46.67% of the IGlar group at week 16 (no significant difference; *p* = 0.397). The total proportion of those with an HbA1c <7% rose from 25.42% to 40.48% with beinaglutide combined with IGlar at week 16 (Figure 2A).

Although FPG decreased over time in the two groups, the proportion of patients with an FPG <7 mmol/L did not differ, being at 55.17% with beinaglutide versus 63.33% with IGlar (*p* = .524) at week 8. Furthermore, the proportion of FBG <7 mmol/L was 62.07% in the beinaglutide group versus 66.67% in the IGlar group at week 16 (*p* = .518). Moreover, the total proportion with an FPG <7 mmol/L increased from 59.32% to 60.04% at week 16 for beinaglutide combined with IGlar (Figure 2B).

### 3.3 Changes in GV indices as measured by CGMS

The IGlar group showed a statistically significant decrease in MG versus baseline for an LS mean of −1.99 (95% CI, −3.52 to −0.46) at 8 weeks. After combined treatment for 8 weeks, both the beinaglutide and IGlar groups had commensurate decreases in MG versus baseline (LS means of −0.91 [95% CI, −1.87 to −0.05]; and −2.15 [95% CI, −3.31 to −0.99], respectively). However, neither treatment significantly reduced LAGE, MAGE, or SDBG (Table 2).

**TABLE 2:** Change in glucose variability after treatments (per‐protocol set).

	8 W				*P*_change_ value	16 W				*P*_change_ value
	Beinaglutide		IGlar			Beinaglutide		Glargine		
	Mean ± SD	Change from baseline, mean (95% CI)	Mean ± SD	Change from baseline, mean (95% CI)		Mean ± SD	Change from baseline, mean (95% CI)	Mean ± SD	Change from baseline, mean (95% CI)	
MG, mmol/L	9.35 ± 2.98	−1.10 (−3.39 to 1.19)	8.52 ± 2.19	−1.99 (−3.52 to −0.46)^a^	.411	9.34 ± 1.84	−0.91 (−1.87 to −0.05)^a^	8.31 ± 2.83	−2.15 (−3.31 to −0.99)^a^	.098
MAGE, mmol/L	5.12 ± 2.20	0.19 (−1.49 to 1.87)	4.06 ± 1.56	−0.00 (−1.17 to 1.17)	.338	4.75 ± 1.86	0.07 (−0.96 to 1.09)	4.18 ± 0.57	−0.37 (−2.05 to 1.30)	.458
LAGE, mmol/L	7.22 ± 2.70	−0.26 (−2.69 to 2.18)	7.05 ± 2.17	0.71 (−0.72 to 2.15)	.989	7.58 ± 2.21	0.17 (−1.34 to 1.67)	6.51 ± 0.64	−0.27 (−1.85 to 1.32)	.219
MODD, mmol/L	2.29 ± 1.06	−0.29 (−1.09 to 0.51)	2.02 ± 1.05	0.06 (−0.71 to 0.72)	.950	2.09 ± 0.55	−0.53 (−0.95 to 0.11)^a^	1.71 ± 0.23	−0.38 (−0.97 to 0.22)	.263
SDBG, mmol/L	2.23 ± 1.11	−0.19 (−0.96 to 0.58)	2.04 ± 0.69	0.10 (−0.54 to 0.74)	.788	2.18 ± 0.69	−0.44 (−1.68 to 0.79)	2.03 ± 0.21	0.05 (−0.61 to 0.71)	.613

*Note*: Data are presented as mean (SD) before and after treatments. Change: values are least‐squares means presented as changes from baseline (95% CI). *P*_*change*_, value changes between groups.

Abbreviations: CI, confidence interval; IGlar, insulin glargine; LAGE, the largest amplitude of glycemic excursions; MAGE, mean amplitude of glycemic excursions; MG, mean glycemic; MODD, mean of daily difference; SDBG, SD of blood glucose.

^a^ Statistical significance achieved between baseline and 8 or 16 weeks. Differences were considered to be statistically significant when the 95% CI did not include 0.

### 3.4 Changes in secondary efficacy outcomes

The changes in secondary efficacy endpoints between baseline and week 8 or week 16 of treatment are shown in Table 3. HbA1c changed by −1.31% (95% CI, 1.69 to −0.94) with beinaglutide and −1.68% (95% CI, −2.05 to −1.30) with IGlar versus baseline. There were no differences in changes in HbA1c between groups, with comparable efficacies (0.33% [95% CI, −0.09 to 0.75]) at 8 weeks. FPG changed by −0.94 mmol/L (95% CI, −1.69 to −0.18) with beinaglutide, and by −2.43 mmol/L (95% CI, −3.60 to −1.28) with IGlar. IGlar was more efficient in lowering FPG than beinaglutide for those with comparable efficacies (−1.57 mmol/L [95% CI, −2.60 to −0.54]). PPG changed by −1.92 mmol/L (95% CI, −3.31 to −0.52) with beinaglutide, and by −3.56 mmol/L (95% CI, −5.59 to −2.14) with IGlar. There were no differences in changes in PPG between groups (1.91 mmol/L [95% CI, −0.01 to 3.83]).

**TABLE 3:** Differences in secondary endpoints from baseline.

	8 W				*P*_change_ value	16 W				*P*_change_ value
	Beinaglutide		IGlar			Beinaglutide		Glargine		
	Mean ± SD	Change from baseline, Mean [95%Cl]	mean ± SD	Change from baseline, Mean [95%Cl]		Mean ± SD	Change from baseline, Mean [95%Cl]	Mean ± SD	Change from baseline, Mean [95%Cl]	
A1C, %	7.87 ± 0.98	−1.31 (−1.69, −0.94)^a^	7.56 ± 0.79	−1.68 (−2.05, −1.30)^a^	0.121	7.87 ± 0.85	−1.35(−1.91, −0.79)^a^	7.63 ± 1.60	−1.62 (−2.33, −1.28)^a^	0.520
FPG, mmol/L	7.94 ± 2.49	−0.94(−1.69, −0.18)^a^	6.34 ± 1.61	−2.43(−3.60, −1.28)^a^	0.003^b^	7.01 ± 1.50	−2.11 (−2.93, −1.28)^a^	6.61 ± 2.40	−2.06 (−3.79, −0.32)^a^	0.556
PPG, mmol/L	14.25 ± 4.07	−1.92(−3.31, −0.52)^a^	12.34 ± 3.72	−3.56 (−5.59, −2.14)^a^	0.051	14.78 ± 3.63	−1.69 (−4.47, 1.08)	13.05 ± 3.07	−3.86 (−6.06, −1.67)^a^	0.146
Weight, kg	67.67 ± 14.49	−2.06 (−2.88, −1.23)^a^	72.50 ± 10.26	0.14 (−0.51, 0.79)	0.001^b^	68.21 ± 15.56	−2.62 (−3.91, −1.34)^a^	71.09 ± 10.13	−0.67 (−1.44, 0.09)	0.010^b^
BMI, kg/m^2^	23.95 ± 3.44	−0.75 (−1.06, −0.44)^a^	26.43 ± 8.91	1.56 (−1.55, 4.67)	0.147	24.07 ± 3.78	−0.96(−1.45, −0.48)^a^	24.71 ± 2.47	−0.24 (−0.51, 0.03)	0.01^b^
Waist, cm	88.40 ± 9.51	−1.53(−2.36, −0.71)^a^	93.33 ± 6.94	−0.25 (−0.90, 0.40)	0.007^b^	88.64 ± 11.76	−1.61 (−5.62, 2.40)	92.85 ± 7.90	−0.17 (−2.00, 1.65)	0.346
Pulse, Beat/min	76.5 ± 6.55	−0.59 (−4.76, 3.59)	77.9 ± 8.83	1.23 (−2.03, 4.50)	0.453	76.8 ± 4.03	−0.57 (−4.56, 3.41)	77.5 ± 8.56	1.54 (−2.35, 5.44)	0.576
SBP, mmHg	118.52 ± 11.16	−6.21 (−11.49, −0.92)^a^	119.67 ± 10.18	−3.53 (−8.87, 1.81)	0.458	106.00 ± 19.23	−7.00 (−14.10, 0.10)	109.10 ± 23.23	−3.68 (−9.30, 1.93)	0.132
DBP, mmHg	76.66 ± 6.56	−2.35 (−5.82, 1.13)	74.00 ± 5.50	−1.53 (−5.31, 2.24)	0.761	74.9 ± 4.94	−1.00 (−4.75, 2.75)	73.86 ± 7.04	−1.91 (−5.26, 1.44)	0.484
Free C−peptides, ng/mL	1.51 ± 0.46	−0.02 (−0.23, 0.19)	1.22 ± 0.77	−0.26 (−0.50,−0.01)^a^	0.06	1.21 ± 0.61	−0.21 (−0.54, 0.12)	1.16 ± 0.62	−0.29(−0.47, −0.12)^a^	0.637
Glucagon, pmol/L	5.63 ± 5.83	−0.06 (−2.17, 2.05)	6.71 ± 5.80	0.32 (−1.25, 1.89)	0.616	5.20 ± 3.48	−0.60 (−1.96, 0.76)	5.67 ± 5.07	−0.71 (−2.74−1.32)	0.886
TC, mmol/L	4.37 ± 0.95	−0.22 (−0.50, 0.06)	4.25 ± 0.92	−0.31 (−0.74. 0.12)	0.615	4.65 ± 0.96	−0.12 (−0.36, −0.12)^a^	4.49 ± 0.84	0.08 (−0.51, 0.35)	0.809
HDL−C, mmol/L	1.27 ± 0.31	0.08 (0.00−0.16)^a^	1.27 ± 0.26	0.03 (−0.05−0.10)	0.368	1.34 ± 0.29	0.09 (0.02, 0.17)^a^	1.26 ± 0.20	0.01 (−0.07, 0.09)	0.116
LDL−C, mmol/L	2.66 ± 0.72	−0.21 (−0.48−0.05)^a^	2.58 ± 0.76	−0.23 (−0.54, 0.07)	0.766	2.88 ± 0.90	−0.14 (−0.30, 0.02)	2.72 ± 0.74	−0.16 (−0.56, 0.23)	0.683
TG, mmol/L	1.60 ± 0.59	0.31 (−0.72,0.05)	1.97 ± 1.94	−0.20 (−0.41, 0.80)	0.169	1.38 ± 0.57	−0.42 (−0.88, 0.04)	2.39 ± 2.98	−0.57 (−0.58, 1.72)	0.121
Uric acid, μmol/L	324.33 ± 72.74	24.64 (4.27, 45.01)	309.31 ± 79.36	17.73 (−1.04, 36.49)	0.486	304.48 ± 78.01	15.27 (−16.38, 46.92)	304.48 ± 78.01	6.54 (−19.99,33.09)	0.668
8−Iso−PGF2α, ng/mL	228.41 (116.92−324.60)	−106.37 (−247.61, 34.87)	170.84 (111.54−328.32)	−27.48 (−151.89, 96.94)	0.312	173.28 (119.56−288.95)	−79.81 (−170.00, 10.38)	152.76 (68.13−254.65)	37.73 (−87.43, 162.89)	0.482
MCP−1, ng/mL	216.5 (165.01−267.51)	15.96 (−5.84, 37.77)	194.36 (177.74−219.38)	−4.45 (−22.01, 13.12)	0.390	230.05 (156.76−258.67)	−2.17 (−25.54, 21.20)	200.38 (167.06−254.98)	9.74 (−12.70, 32.18)	0.603
hs−CRP, mg/dL	587.32 (268.77−127.32)	−963.21 (−1929.58, 3.16)	579.59 (280.99−122.27)	−151.39 (−611.75, 308.97)	0.069	6215.79 (344.07−162.18)	−885.48 (−1977.56, 206.60)	557.61 (233.89−999.62)	1545.40 (−2593.95, 5684.75)	0.437
Urinary albumin, mg/L	19.55 (6.81− 61.33)	−1.58 (−17.38, 14.21)	11.40 (3.84−20.90)	2.36 (−10.80, 15.53)	0.666	21.10 (12.40−82.60)	68.67 (−93.91, 231.26)	9.52 (6.79−22.90)	9.85 (−17.83, 37.54)	0.142

*Note:* Data after treatment are presented as mean (SD) or median (range interquartile). Change: values are least‐squares means presented as changes from baseline (95% CI). *P*_change_: Value changes between groups.

Abbreviations: BMI, Body mass index; CRP, C‐reactive protein; DBP, Diastolic blood pressure; HDL‐C, High‐density lipoprotein cholesterol; LDL‐C, Low‐density lipoprotein cholesterol; MCP‐1, Monocyte chemoattractant protein‐1; TG, Triglycerides; TC, Total cholesterol; SBP, Systolic blood pressure.

^a.^ Statistical significance achieved from baseline. Differences were considered to be statistically significant when the 95% CI did not include 0.

^b^ Significant differences between the beinaglutide group and the IGlar group.

Compared with baseline, participants treated with beinaglutide showed a 2.06 kg (95% CI, 1.23–2.88) decrease in body weight, while participants treated with IGlar did not exhibit a change in body weight of more than 0.14 kg (95% CI, −1.51 to 0.79). Beinaglutide was thus more efficient in decreasing body weight than IGlar in those with comparable efficacies (−2.25 kg [95% CI, −3.32 to − 1.22], *p* < .001).

Similarly, compared with baseline, participants taking beinaglutide had a decreased BMI of 0.75 kg/m^2^ (95% CI, 0.44–1.06), whereas participants taking IGlar did not, although there were no differences in the changes in BMI between groups (−2.30 kg/m^2^ [95% CI, −5.44 to 0.84]).

Importantly, compared with baseline, participants treated with beinaglutide had a reduced waist circumference of 1.53 cm (95% CI, 0.71–2.36), whereas patients taking IGlar reduced their waist circumference only by 0.25 cm (95% CI, −0.90 to 0.40). Beinaglutide was thus more efficient in reducing waist circumference than IGlar (1.45 cm [95% CI, 0.41–2.49]).

Systolic blood pressure (SBP) decreased in participants using beinaglutide by 6.21 mm Hg (95% CI, 0.92–11.49), whereas participants using IGlar did not show a similar diminution (3.53 mm Hg [95% CI, −1.81 to 8.87]). However, there were no differences in changes in SBP or diastolic blood pressure (DBP) between groups, and the comparable efficacies (−1.70 mm Hg [95% CI, −6.25 to 2.85], and 1.93 mm Hg [95% CI, −1.11 to 4.96], respectively).

Regarding the measured lipid concentrations, HDL‐C increased by 0.08 mmol/L (95% CI, 0.00–0.16), and although LDL‐C decreased by 0.21 mmol/L (95% CI, 0.05–0.48) at week 8 in the beinaglutide group compared with baseline, the HDL‐C and LDL‐C did not change in the IGlar group.

After taking beinaglutide combined with IGlar for 8 weeks, both the beinaglutide and IGlar groups showed further reduction in HbA1c at 16 weeks versus baseline (1.35% [95% CI, 0.79–1.91], and 1.62% [95% CI, 1.28–2.33], respectively), with comparable efficacies (−0.26% [95% CI, −0.54 to 1.05]) at week 16. The decrease in FPG was not significant between the two groups (2.11 mmol/L [95% CI, 1.28–2.93], and 2.06 mmol/L [95% CI, 0.32–3.79], respectively), with comparable efficacies (0.37 mmol/L [95% CI, −0.86 to 1.61]) at week 16.

Two‐hour PPG was not altered in the beinaglutide group at 16 weeks versus baseline (−1.69 mmol/L [95% CI, −4.47 to 1.08]), whereas 2‐h PPG changed in the IGlar groups at 16 weeks versus baseline (−3.86 mmol/L [95% CI, −6.06 to −1.67]). We noted no differences in changes in PPG between groups (1.69 mmol/L [95% CI, −0.62 to 4.01]).

Body weight was diminished by 2.62 kg (95% CI, 1.34–3.91) in the beinaglutide group but was not affected in the IGlar group (−0.67 kg [95% CI, −1.44 to 0.09]) at 16 weeks compared with baseline. Participants in the beinaglutide group lost more weight than those in the IGlar group with comparable efficacies (1.96 kg [95% CI, 0.51–3.41]). Similarly, at 16 weeks compared with baseline, participants reduced BMI by 0.96 kg/m^2^ (95% CI, 0.48–1.45) in the beinaglutide group, whereas there was no change in the IGlar group (0.24 kg/m^2^ [95% CI, −0.03 to 0.51]), with comparable efficacies (− 0.72 kg/m^2^ [95% CI, −1.26 to −0.19], *p* = .010). There was also no change in waist circumference at 16 weeks versus baseline between the beinaglutide and IGlar groups, with comparable efficacies (−2.06 cm [95% CI, −6.40 to 2.28]).

Compared with baseline, SBP did not change in either group at 16 weeks (7.00 mm Hg [95% CI, −0.10 to 14.10], and 3.68 mm Hg [95% CI, −1.36 to 9.30], respectively). There were also no differences in changes in SBP and DBP between groups, and they had comparable efficacies (−4.15 mm Hg [95% CI, −9.60 to 1.31], and 1.12 mm Hg [95% CI, −2.09 to 4.33], respectively).

With regard to lipid concentrations, HDL‐C increased by 0.09 mmol/L (95% CI, 0.02–0.17) in the beinaglutide group, with no change in the IGlar group at 16 weeks.

Inflammatory factors, including 8‐iso‐PGF2α, MCP‐1, and Hs‐CRP, as well as urinary albumin did not change in either group at 8 or 16 weeks compared with baseline.

### 3.5 Safety and tolerability

Both treatments were well tolerated and no SAEs were observed. There were 32 AEs in 20 (57.14%) participants in the beinaglutide group and 30 AEs in 22(66.67%) participants in the IGlar group, and of these, 29 AEs in 16 (45.71%) participants in the beinaglutide group and 26 AEs in 18 (54.54%) participants in the IGlar group were related to the study drug. Four participants in the beinaglutide group and one participant in the IGlar group withdrew from the study due to AEs. There was no difference in reported hypoglycemic events between the two groups (5 [14.29%] vs. 9 [27.27%], *p* = .186). The beinaglutide group exhibited a greater incidence of nausea, discomfort, and gastrointestinal side effects than the IGlar groups (9 [25.71%] vs. 2 [6.1%], *p* = .028). Physical examinations that included vital signs, electrocardiograms, routine laboratory chemistry, and hematologic tests did not reveal any clinically significant changes between groups.

### 3.6 Effect of fasting C‐peptide ≥0.9 or <0.9 ng/mL on change in glucose after treatments

Of all 59 participants who completed this study, there were 11 participants with a fasting C‐peptide <0.9 ng/mL, including 5 in the beinaglutide group and 6 in the IGlar group. In addition, there were 48 participants with a fasting C‐peptide ≥0.9 ng/mL, including 24 in the beinaglutide group and 24 in the IGlar group. With respect to a fasting C‐peptide of 0.9 ng/mL, we divided the participants into a fasting C‐peptide ≥0.9 ng/mL group and a fasting C‐peptide <0.9 ng/mL group.

**FIGURE 3:**
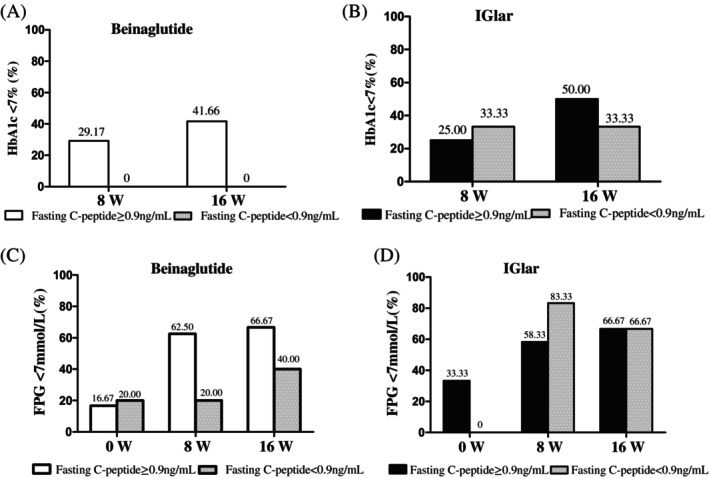
Proportions of the participants reaching the target with fasting C‐peptides ≥0.9 or <0.9 ng/mL. (A) Glycated hemoglobin (HbA1c) control achieved target at week 8 and week 16 in the beinaglutide group when fasting C‐peptides were ≥0.9 or <0.9 ng/mL. (B) HbA1c control achieved target at week 8 and week 16 in the IGlar group when fasting C‐peptide ≥0.9 or <0.9 ng/mL. (C) Fasting plasma glucose (FPG) value control achieved target at baseline, week 8, and week 16 in the beinaglutide group when fasting C‐peptides were ≥0.9 or <0.9 ng/mL. (D) Fasting plasma glucose (FPG) value control achieved target at baseline, week 8, and week 16 in the IGlar group when fasting C‐peptides were ≥0.9 or <0.9 ng/mL. Data were presented as *n* (%). IGlar.

The proportions of patients with an HbA1c <7% did not differ between the beinaglutide and the IGlar groups at week 8 and week 16 with a fasting C‐peptide ≥0.9 ng/mL. However, in the beinaglutide group, 7 participants reached the HbA1c target at 8 weeks and 10 of participants reached the target at 16 weeks; all of them had fasting C‐peptide ≥0.9 ng/mL, and none of the participants with fasting C‐peptide <0.9 ng/mL at 8 weeks or 16 weeks reached the target (Figure 3A). In the IGlar group, 6 of 24 participants with a fasting C‐peptide ≥0.9 ng/mL reached the HbA1c target at 8 weeks, 10 reached the HbA1c target at 16 weeks, 2 of 6 participants with a fasting C‐peptide <0.9 ng/mL reached the target at 8 weeks, and 4 of 6 reached the target at 16 weeks (Figure 3B).

Regarding FPG, in the beinaglutide group, 15 of 24 (62.5%) participants with a fasting C‐peptide ≥0.9 ng/mL reached an FPG <7 mmol/L at 8 weeks, whereas 16 of 24 (66.67%) of those participants reached an FPG <7 mmol/L at 16 weeks; none of the participants with a fasting C‐peptide <0.9 ng/mL reached the target of FPG <7 mmol/L at 8 weeks (excepted for the participant whose FPG was <7 mmol/L at baseline) (Figure 3C). In the IGlar group, 5 of 6 participants with a fasting C‐peptide <0.9 ng/mL attained the target of FPG <7 mmol/L at 8 weeks, and 4 of 6 of them reached the target of FBG <7 mmol/L (Figure 3D).

Among 48 participants with fasting C‐peptide ≥0.9 ng/mL, FPG was lowered by 1.40 mmol/L (95% CI, 0.96–1.87) with beinaglutide and by 1.70 mmol/L (95% CI, 0.96–2.43) with IGlar at week 8. We noted no differences between the two groups in the change in FBG level, meaning they had comparable efficacies (−0.60 mmol/L [95% CI, −0.25 to 1.44]).

## 4 DISCUSSION

In this randomized controlled trial, we aimed to compare the efficacy and safety of beinaglutide versus IGlar, as well as the combination of beinaglutide and IGlar, in Chinese patients with T2DM whose blood glucose levels were inadequately controlled with oral antidiabetic drugs. We ascertained that both beinaglutide or IGlar alone, or the two drugs combined, effectively improved glucometabolism. As expected, both beinaglutide and IGlar were effective in reaching HbA1c and FPG targets after treatment for 8 weeks, and the combination of beinaglutide and IGlar augmented the proportions of patients reaching the HbA1c target at 16 weeks of treatment. Neither group experienced serious hypoglycemia, and although both showed comparable hypoglycemic events, beinaglutide manifested more numerous gastrointestinal side effects than IGlar. In addition, beinaglutide led to reduced body weight and waist circumference, whereas IGlar did not accomplish these end points. To the best of our knowledge, this is the first‐ever analysis to compare the hypoglycemic efficacy of beinaglutide versus IGlar or beinaglutide combined with IGlar.

Our results revealed that beinaglutide (0.1–0.2 mg through in a subcutaneous injection three times per day) exerted a hypoglycemic effect in patients who showed inadequate control in reducing HbA1c, FPG, and PPG with oral antidiabetic drugs at 8 weeks. These data were consistent with previous studies on the same end points,^18^, ^19^ thus confirming the efficacy and the safety of beinaglutide in glycemic control. This study also provided evidence for the hypoglycemic effect of beinaglutide. Among the different combined hypoglycemic treatment regimens, combined therapy with GLP‐1RA and basal insulin reflected the advantages of a lower hypoglycemic risk and more weight reduction with noninferior potency of glycemic control; this enables the achievement of the ideal goal in diabetic treatment robust glycemic control with no increased hypoglycemia or weight gain.^28^ This drug combination is thus a potential therapeutic strategy that could improve the management of patients with T2DM, and our results support this view. After patients were administered combined beinaglutide and IGlar, the proportion reaching their HbA1c target increased from 25.42% with beinaglutide alone to 40.68%, and the proportion reaching their HbA1c target increased from 26.67% with IGlar alone to 46.67% at 16 weeks, indicating that the combined regimen of the two agents can further improve blood glucose control. Chronic hyperglycemia leads to diminished glucose‐stimulated insulin secretion and increased insulin resistance, and resolving this glucose toxicity is a key to the treatment of T2DM, as the decline in insulin secretion and insulin sensitivity will lead to further hyperglycemia. The combination of basal insulin and GLP‐1RA can thus resolve the two issues of hyperglycemia in T2DM patients. Due to the advantages of this combination, several products containing fixed‐ratio combinations of basal insulin and GLP‐1RA have entered the clinical use.^24^, ^29^

An important finding of our study was that beinaglutide provided efficacy in blood pressure control and a reduction in SBP. Hypertension is a common risk factor of macrovascular complications, and diabetic patients with these conditions are at even higher risk for developing cardiovascular disease.^30^, ^31^, ^32^ Lower BP reduces the risk of diabetes complications, and an average SBP decrease of 6 mm Hg in T2DM patients results in a reduction in the risks of coronary heart disease, cerebrovascular disease, and kidney disease of 18%, 6%, and 21%, respectively.^8^, ^33^ Participants taking beinaglutide showed superior control of SBP, and this suggests its potential use as a valuable and worthy alternative treatment for patients without good glucose control in terms of cardiovascular disease (CVD) benefits.

We determined that beinaglutide provided efficacy in improving lipid metabolism by increasing of HDL‐C, as well as decreasing LDL‐C. Patients with diabetes exhibit dyslipidemia characterized by an enhanced production of LDL‐C and decreased HDL‐C production.^34^, ^35^ Dyslipidemia constitutes a major risk factor for the development of CVDs in T2DM patients, with a 1 mmol/L reduction in LDL‐C correlating with a 21% reduction in major adverse cardiovascular events.^36^ A Japanese study showed that GLP‐1RA attenuated LDL‐C level but not HDL‐C or TG during follow‐up.^37^ In a review, authors elaborated the mechanism underlying GLP‐1's regulation of lipid metabolism disorders in T2DM patients and indicated that GLP‐1 regulates lipid‐metabolism disorders and provides additional benefits beyond hypoglycemic therapy.^38^ Our results also revealed that beinaglutide improved lipid metabolism in T2DM patients similar to the actions of other GLP‐1RAs.

Our results showed that there was a more effective lowering of FPG levels in the IGlar group than in the beinaglutide group (LS mean difference −1.57 mmol/L [95% CI, − 2.60 to −0.54]) after 8 weeks of treatment. After analyzing our data, we found that all of the patients in the beinaglutide group whose FPG reached the target after 8 weeks of treatment had baseline C‐peptides ≥0.9 ng/mL. In patients with a fasting C‐peptides ≥0.9 ng/mL, the differences between the two groups in terms of lowering FPG levels disappeared (−0.60 mmol/L [95% CI, −0.25 to 1.44]). These results indicate that the fasting C‐peptide levels of patients may influence the hypoglycemic effects of beinaglutide, and there have been reports on the relationship between beta‐cell function in patients and the hypoglycemic effects of GLP‐1RAs. A patient‐level, pooled analysis of the Semaglutide Unabated Sustainability in Treatment of Type 2 Diabetes (SUSTAIN) 1–5 trials showed that the reduction in HbA1c due to semaglutide was related to baseline characteristics that included beta‐cell function. Patients were divided into three groups according to homeostatic model assessment of β‐cell function (HOMA‐β) tertile, and a reduction in HbA1c was observed in all baseline HOMA‐β tertiles, with the reduction decreasing from low to high HOMA‐β tertiles.^39^ There was also a significant correlation between the area under the C‐peptide immunoreactivity curve and changes in HbA1c. We recommend beta‐cell function be evaluated for the effective introduction of GLP‐1RA to patients with diabetes and that a determination be made as to whether to convert to or add GLP‐1RA treatment in patients with T2DM.^40^ A meta‐analysis of 620 people indicated that clinical markers of low beta‐cell function were associated with reduced glycemic response to GLP‐1RA therapy. C‐peptide levels and islet autoantibodies also represent potential biomarkers for the stratification of GLP‐1RA therapy in insulin‐treated diabetes.^41^

Our results also revealed that there was no difference between the two groups in the achievement targets for HbA1c and FPG at 8 weeks, and no patients with fasting C‐peptides <0.9 ng/mL achieved the targeted HbA1c and FPG at 8 weeks after using beinaglutide. However, the achievement targets for HbA1c and FPG might not have been influenced by C‐peptide levels in patients with IGlar.

Although the authors of several guidelines recommend that GLP‐1RA as the first‐line treatment of high risk for ASCVD, it is still necessary to evaluate the beta‐cell function of patients in terms of determining whether to use GLP‐1RA as a hypoglycemic therapy.

There were several limitations to the current study. First, we enrolled a limited sample size, and hence additional studies of adequate sample size are necessary to confirm our current findings. Second, due to funding limitations, our follow‐up time was only 16 weeks, including 8 weeks of beinaglutide or glargine treatment and 8 weeks of combined treatment, such that the treatment duration for each regimen was relatively short. We acknowledge this to be a major limitation to our study and consider it a possible factor underlying our results where beinaglutide did not significantly change major GV indices such as MAGE, LAGE, MODD, or SDBG. Furthermore, due to the short follow‐up time, the initial dosage of beinaglutide was 0.1 mg, 3/day, with the dosage increasing to 0.2 mg, 3/day after 1 week of treatment. Some patients could not tolerate this increase, and this resulted in a higher dropout rate in the beinaglutide group. We suggest that the dose titration of beinaglutide in the clinical setting be increased according to the individual response of the patients.

## 5 CONCLUSIONS

Beinaglutide reduces blood glucose, weight, and SBP, and improves lipid metabolism. The fasting C‐peptide levels of patients were related to the hypoglycemic effect of beinaglutide, and C‐peptide should therefore be evaluated when using or converting to beinaglutide. However, because beinaglutide is a new GLP‐1RA, clinical reports on its use are relatively limited, and more research data are needed to verify our results.

## AUTHOR CONTRIBUTIONS

Qiuhe Ji and Li Wang conceived and designed the study. Wenjuan Yang, Jianrong Liu, Xinxi Huang, Yujie Fang, Jie Ming, Jingbo Lai, and Jianfang Fu contributed to the data collection. Xiangyang Liu, Wenjuan Yang, Jianrong Liu, Jingbo Lai, Jianfang Fu, and Li Wang contributed to the data extraction and interpreted the results. Xiangyang Liu, Jingbo Lai, Jianfang Fu, and Li Wang performed the data analysis. Xiangyang Liu, Qiuhe Ji, and Li Wang wrote the first draft. All authors read and approved the final manuscript.

## FUNDING INFORMATION

This work was supported by a grant from The Bethune Foundation and a grant from the National Natural Science Foundation of China (Grant No. 31571415).

## DISCLOSURE

The authors have no conflicts of interest to declare.

## Data Availability

The clinical data sets of the trial are not publicly available to ensure compliance with the ethical approval obtained for the study but can be obtained from the corresponding authors upon reasonable request.
